# Abnormal pattern of vitamin D receptor-associated genes and lncRNAs in patients with bipolar disorder

**DOI:** 10.1186/s12888-022-03811-8

**Published:** 2022-03-12

**Authors:** Reyhane Eghtedarian, Soudeh Ghafouri-Fard, Hamid Bouraghi, Bashdar Mahmud Hussen, Shahram Arsang-Jang, Mohammad Taheri

**Affiliations:** 1 Department of Medical Genetics, School of Medicine, Shahid Beheshti University of Medical Sciences, Tehran, Iran; 2 Men’s Health and Reproductive Health Research Center, Shahid Beheshti University of Medical Sciences, Tehran, Iran; 3 Department of Health Information Technology, School of Paramedical Sciences, Hamadan University of Medical Sciences, Hamadan, Iran; 4 Department of Pharmacognosy, College of Pharmacy, Hawler Medical University, Erbil, Kurdistan Region Iraq; 5 Cancer Gene Therapy Research Center, Zanjan University of Medical Science, Zanjan, Iran; 6 Skull Base Research Center, Loghman Hakim Hospital, Shahid Beheshti University of Medical Sciences, Tehran, Iran; 7 Institute of Human Genetics, Jena University Hospital, 07740 Jena, Germany

**Keywords:** Bipolar disorder, VDR, MALAT1, SNHG6, LINC00511, LINC00346

## Abstract

**Background:**

Bipolar disorder (BD) is a multifactorial condition. Several signaling pathways affect development of this disorder. With the purpose of exploring the role of vitamin D receptor (VDR) signaling in this disorder, we measured expression of selected mRNA coding genes and long non-coding RNAs (lncRNAs) in this pathway in patients versus normal subjects.

**Methods:**

We measured expression of VDR-associated lncRNAs and mRNAs (*SNHG6*, *MALAT1*, *Linc00511*, *Linc00346*, *VDR* and *CYP27B1*) in the peripheral blood of BD patients vs. healthy individuals.

**Results:**

Expression of *SNHG6* was significantly higher in cases vs. controls (Posterior beta = 1.29, *P* value < 0.0001. Subgroup analysis by sex revealed significant results in both subgroups (*P* value < 0.0001 and *P* value = 0.023 for males and females, respectively). Expression of *CYP27B1* was up-regulated in cases vs. controls (Posterior beta = 0.415, *P* < 0.0001). Such pattern was also detected among males (*P* < 0.0001), but not females (*P* = 0.419). Similarly, *MALAT1* and *Linc00346* were up-regulated in total cases vs. controls (Posterior beta = 0.694, *P* < 0.0001 and Posterior beta = 0.4, *P* = 0.012, respectively) and in male cases compared with male controls (Posterior beta = 0.712, *P* < 0.0001 and Posterior beta = 0.41, *P* value = 0.038, respectively). Expression of *VDR* was up-regulated in total cases compared with controls (Posterior beta = 0.683, *P* value = 0.001). Finally, expression of *Linc00511* was not different between groups. *MALAT1*, *SNHG6*, *CYP27B1*, *VDR* and *Linc00346* had AUC values of 0.95, 0.94, 0.91, 0.85 and 0.83 in differentiation of male patients from controls, respectively.

**Conclusion:**

The current study suggests VDR-associated genes as possible markers for BD.

## Introduction

Bipolar disorder (BD) is a multicomponent disorder. In addition to incidences of severe mood disturbances and neuropsychological abnormalities, some immunological and physiological alterations have been noted in BD [1, 2]. BD is considered as a major source of disability throughout the world [3] being correlated with elevated risk of premature death from both suicide and comorbid conditions [4, 5]. Although the main etiology BD is not clear, several genetic factors, essential developmental pathways, and gene–environment interacting factors have been identified that presumably participate in the development of BD [6]. Vitamin D receptor (VDR) signaling is among putative contributors in this regard. For instance, BD in adolescence has been linked with high concentrations of vitamin D-binding protein [7]. In addition, *VDR* FokI genotype has been shown to influence risk of BD through modulation of expression of dopamine D1 receptor [8]. Moreover, treatment with vitamin D3 supplement has been shown to improve mood symptoms and brain neurochemistry in adolescent mania patients [9]. Through binding with VDR, vitamin D has been shown to affect monoamine metabolism by influencing the hypothalamic–pituitary–adrenal axis [10–12]. VDR can also influence nuclear transcription and modulate levels of the dopamine receptor gene [13]. The importance of VDR in the modulation of function of mitochondria and metabolic pathways of lipids [14] further supports the role of this molecule in the pathoetiology of neuropsychiatric disorders such as BD.

Numerous VDR response elements have been identified in the genomic regions such as those corresponding to long non-coding RNAs (lncRNAs) [15]. LncRNAs are a group of non-coding RNAs with sizes more than 200 nucleotides. Although these transcripts have several similar features with mRNAs, they do not produce proteins. Yet, they regulate expression of genes through various mechanisms including epigenetic modifications [16]. We have designed an *in-silico* approach to recognize lncRNAs which are functionally linked with VDR signaling. This approach has been first used in the context of breast cancer and led to identification of *MALAT1*, *SNHG6*, *LINC00511* and *LINC00346* as VDR-related lncRNAs [17]. The observed correlations between these lncRNAs and VDR in breast tissues provided further clues for their relevance with VDR signaling [17]. Since transcriptomic data of lncRNAs in neuropsychiatric disorders were not sufficient to perform similar approach to find VDR-associated lncRNAs in these disorders, we suppose that the observed associations between lncRNAs and VDR in breast cancer can also been detected in these disorders. Subsequent expression assays have verified abnormal expression of a number of these transcripts in schizophrenia [18] and provided supports for our assumption. In the present work, we aimed at appraisal of expression of *VDR*, these lncRNAs and vitamin D activating enzyme 1-alpha-hydroxylase (*CYP27B1*) in the circulation of patients with BD versus normal individuals to appraise the association between these genes and occurrence of BD. CYP27B1 was selected as an example of VDR-related enzymes due to its possible role in the pathoetiology of neurological disorders [19].

## Materials and methods

### Bioinformatics methods

For the purpose of identification of VDR-related lncRNAs, the following tools were used: 1) Identification of lncRNAs that act upstream of VDR using LncRNA2Target tool (http://www.lncrna2target.org), This online tool facilitates identification of targets of a certain lncRNA and identification of lncRNAs that target a certain gene. 2) Identification of mutations in these lncRNAs or VDR-related genes through assessment of genomic sequencing datasets using cBioPortal for Cancer Genomics (http://cbioportal.org), and 3) Recognition of lncRNAs that are targets of genomic alterations in cancer tissues the Catalog of Somatic Mutations in Cancer (COSMIC).

### Enlisted persons

A total of 50 type I BD patients and 50 controls were enrolled in the in the present research. Patients were diagnosed using the DSM-5 criteria [20]. Patients were enlisted from Farshchian Hospital (Hamadan, Iran) and Imam Hossein (Tehran, Iran) during February 2018-December 2020. All patients had been taking Carbamazepine (200 mg 2 times a day). With the aim of decreasing the diversity of patients’ cohort, we excluded those who were under treatment with other drugs. During the study period, we consecutively recruited all patients having the mentioned criteria (15 females and 35 males). All patients had stable mood as diagnosed by structured/semistructured diagnostic tools. Then, we recruited healthy persons with the same male/female ratio to serve as controls. Controls had no psychiatric disorder. Former history of psychiatric or neurodegenerative disorders, intellectual disabilities, cancer or infection were regarded as criteria for exclusion of subjects from this study group. Cases and controls were also matched according to ethnicity, education level and socioeconomic parameters. None of them had undergone any treatment procedure. The study protocol was approved by the ethical committee of Shahid Beheshti University of Medical Sciences (IR.SBMU.MSP.REC.1399.290) and all methods were performed in accordance with the relevant guidelines and regulations. Written consent forms were signed by all study participants.

### Experiments

Blood samples were collected from BD patients and controls in EDTA-containing tubes. Total RNA was retrieved from these samples using GeneAll RNA extraction kit (Seoul, South Korea). Afterwards, about 2 µg of RNA was converted to complementary DNA using the BioFac kit (Seoul, South Korea). Levels of *VDR*-associated genes were measured in all samples in relation with expression of *B2M* gene using the RealQ Plus 2 × Master Mix (Amplicon, Denmark). Primers were designed based on MIQUE-guidelines (https://doi.org/10.1373/clinchem.2008.112797) and were used in our previous study [17]. Specificity of primers was assessed using NCBI primer designing tool (https://www.ncbi.nlm.nih.gov/tools/primer-blast/). Moreover, PCR products were run on 2% agarose gel to rule out the presence of extra bands. Table 1 demonstrates the information about primers sequences and amplicons.

**Table 1:** Information about primers and PCR products

Gene Name	Sequence 5’ → 3’	Size of the amplicon (bp)
*B2M*	F: AGATGAGTATGCCTGCCGTG R: GCGGCATCTTCAAACCTCCA	105
*VDR*	F: TGGCTTTCACTTCAATGCTATGA R: CGTCGGTTGTCCTTGGTGAT	126
*CYP27B1*	F: CCCAGATCCTAACACATTTTGAGG R: AAAGGGTGATGATGACAGTCTCTTTC	152
*MALAT1*	F: GACGGAGGTTGAGATGAAGC R: ATTCGGGGCTCTGTAGTCCT	84
*SNHG6*	F: AGGGAGGAAGAAGCGCGAA R: TCGCAGAGCCCAGCTACG	85
*LINC00346*	F: TGCCCTGGACATTCATGGAC R: CTGGACAAGCCCACTCTAGC	150
*LINC00511*	F: TCCCACCAGGAAGTTTAGCAG R: GCCTCTCAAGAGGTGGTCC	87

### Statistical methods

To assess possible differences in expression levels between groups, we used Bayesian quantile regression model with asymmetric Laplace prior distribution. This model was used because of distribution patterns of gene expression data. By using the Bayesian models, we can also provide more accurate estimates by including the prior knowledge about the parameters of interest. One of the major advantages of the Bayesian approach is its extreme flexibility. Bayesian methods provide the opportunity for fitting realistic models according to complex data sets with measurement errors, censored or missing observations, multilevel or serial correlation structures. It also provides an accurate way to combine prior information to a large data set within a theoretical framework [21].

Data were assessed using the Stan, ‘ggplot2’, ‘brms’,’ bestNormalize’, and pROC packages in the R v.4 environment. Expressions of genes in each sample were quantified using the Efficiency adjusted Ct of the normalizer gene—Efficiency adjusted Ct of the target gene (comparative –delta Ct method). Moreover, the fold change of the test sample relative to the control sample was calculated using the 2^−delta delta^ Ct formula. *P* value was appraised from frequentist method using quantile regression and mixed effects models. Receiver operating characteristic (ROC) curves were illustrated to assess the diagnostic power of gene for differentiation between cases and healthy subjects. Youden’s J parameter was measured to find the optimum threshold. This was tested against the original sample.

## Results

### General data

Each study subgroup included 15 females and 35 males. Mean, standard deviation and range of age parameter among cases and controls were 36.5 ± 9.32 (17–56) and 33.62 ± 8.59 (14–52), respectively (Table 2).

**Table 2:** General data of cases and controls

Groups	Clinical/demographic factors		Values
Case	Gender	Male	35
		Female	15
	Age (mean ± SD (range))		36.5 ± 9.32 (17–56)
	Age at onset (mean ± SD (range))		32.64 ± 8.04 (15–48)
	Disease duration (mean ± SD (range))		3.86 ± 2.66 (1–14)
Control	Gender	Male	35
		Female	15
	Age (mean ± SD (range))		33.62 ± 8.59 (14–52)

### Expression assays

**Fig. 1:**
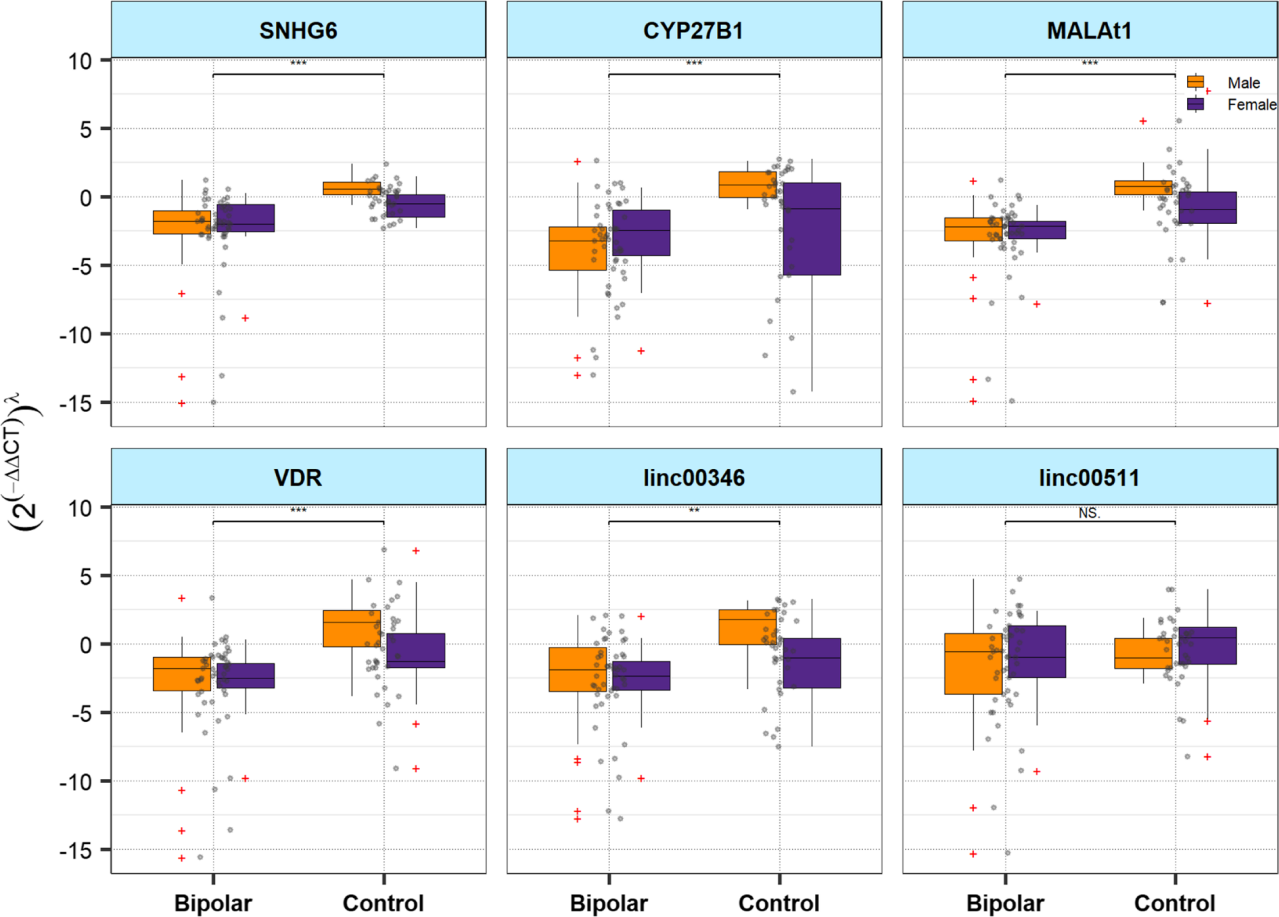
Relative expressions of VDR-associated genes in patients and controls based on their gender (ʎ: Power transformation value estimated from Box-cox or Yeo-Johnson methods; Outliers are shown by red crosses; Upper quartile, median and lower quartile levels are shown. NS: not significant, *: *P* < 0.05, **: *P* < 0.01)

Figure 1 demonstrates relative expressions of VDR-associated genes in cases and controls based on their gender.

**Fig. 2:**
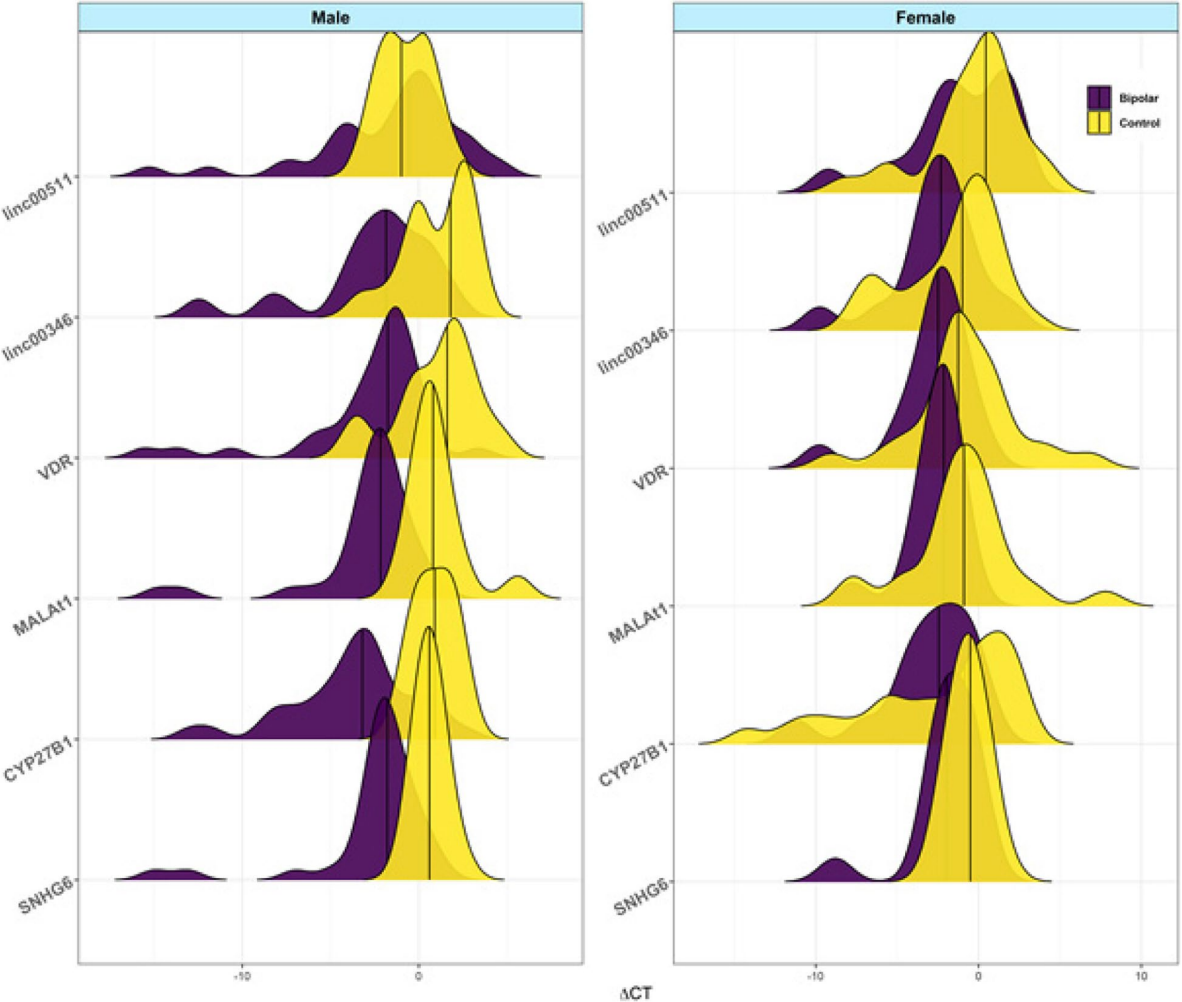
Distribution of delta Ct values in indifferent groups (cases and controls) and different sexes (females and males)

Distribution of delta Ct values in indifferent groups (cases and controls) and different sexes (females and males) are shown in Fig. 2. These plots were depicted using the mentioned packages in R environment to further depict the degree of overlap between expression values of genes in study subgroups.

Expression of *SNHG6* was meaningfully higher in cases vs. controls (Posterior beta = 1.29, *P* value < 0.0001. The interaction between sex and group was significant for this lncRNA (*P* = 0.04). Subgroup analysis by sex revealed significant results in both subgroups (*P* value < 0.0001 and *P* value = 0.023 for males and females, respectively).

Expression of *CYP27B1* was up-regulated in cases vs. healthy subjects (Posterior beta = 0.415, *P* < 0.0001). Such pattern was also detected among males (*P* < 0.0001), but not females (*P* = 0.419). Similarly, *MALAT1* and *Linc00346* were up-regulated in total cases vs. controls (Posterior beta = 0.694, *P* < 0.0001 and Posterior beta = 0.4, *P* = 0.012, respectively) and in male cases compared with male controls (Posterior beta = 0.712, *P* < 0.0001 and Posterior beta = 0.41, *P* value = 0.038, respectively). Expression of *VDR* was up-regulated in total cases compared with controls (Posterior beta = 0.683, *P* value = 0.001). Finally, expression of *Linc00511* was not different between groups (Table 3). Using Post-hoc power calculator, the study power has been calculated to be more than 80% (α = 0.05).

**Table 3:** The association between relative expressions of VDR-associated genes and BD based on the results of Bayesian Quantile regression model. When the interaction effects were significant, subgroup analysis by sex was performed to interpret the associations

Comparisons		Posterior Beta of (2^(−ddct)^)^ʎ^	SE	Adjusted *P*-value^a^	95% Credible Intervals for Beta
*SNHG6*					
Total	Patients vs. healthy subjects	1.299	0.2	< 0.0001	[0.89, 1.7]
	Females vs. Males	0.016	0.08	0.736	[-0.14, 0.18]
	Age (years)	-0.001	0.003	> 0.999	[-0.01, 0.005]
	Group ^a^ Sex	-0.539	0.13	0.04	[-0.78, -0.29]
Male	Patients vs. healthy subjects	0.772	0.1	< 0.0001	[0.58, 0.98]
	Age	-0.001	0.004	0.972	[-0.01, 0.01]
Female	Patients vs. healthy subjects	0.223	0.1	0.023	[0.04, 0.43]
	Age	-0.001	0.005	0.967	[-0.01, 0.01]
*CYP27B1*					
Total	Patients vs. healthy subjects	0.415	0.07	< 0.0001	[0.28, 0.56]
	Females vs. Males	0.083	0.07	0.08	[-0.05, 0.21]
	Age (years)	0.003	0.003	0.19	[-0.002, 0.01]
	Group ^a^ Sex	-0.289	0.12	0.046	[-0.52, -0.06]
Male	Patients vs. healthy subjects	0.404	0.06	< 0.0001	[0.28, 0.53]
	Age	0.002	0.003	0.717	[-0.01, 0.01]
Female	Patients vs. healthy subjects	0.189	0.1	0.419	[-0.05, 0.36]
	Age	0.0001	0.006	0.178	[-0.01, 0.02]
*MALAT1*					
Total	Patients vs. healthy subjects	0.694	0.1	< 0.0001	[0.5, 0.88]
	Females vs. Males	-0.019	0.08	0.624	[-0.18, 0.14]
	Age (years)	-0.003	0.003	0.294	[-0.01, 0.004]
	Group ^a^ Sex	-0.501	0.14	0.021	[-0.77, -0.23]
Male	Patients vs. healthy subjects	0.712	0.1	< 0.0001	[0.52, 0.9]
	Age	-0.002	0.004	0.439	[-0.01, 0.01]
Female	Patients vs. healthy subjects	0.186	0.13	0.096	[-0.07, 0.44]
	Age	-0.003	0.007	0.541	[-0.02, 0.01]
*Linc00346*					
Total	Patients vs. healthy subjects	0.4	0.09	0.012	[0.21, 0.56]
	Females vs. Males	-0.041	0.07	0.078	[-0.17, 0.1]
	Age (years)	0.003	0.001	0.588	[-0.003, 0.01]
	Group ^a^ Sex	-0.251	0.12	0.012	[-0.48, -0.02]
Male	Patients vs. healthy subjects	0.41	0.08	0.038	[0.23, 0.56]
	Age	0.007	0.001	0.238	[-0.003, 0.02]
Female	Patients vs. healthy subjects	0.147	0.08	0.17	[-0.01, 0.3]
	Age	0.002	0.001	0.695	[-0.01, 0.01]
*VDR*					
Total	Patients vs. healthy subjects	0.683	0.21	0.001	[0.29, 1.1]
	Females vs. Males	-0.088	0.12	0.292	[-0.33, 0.17]
	Age (years)	-0.006	0.01	0.596	[-0.02, 0.01]
	Group ^a^ Sex	-0.45	0.24	0.16	[-0.93, 0.004]
*Linc00511*					
Total	Patients vs. healthy subjects	0.019	0.08	0.920	[-0.13, 0.18]
	Females vs. Males	0.015	0.1	0.175	[-0.18, 0.22]
	Age (years)	0.007	0.001	0.006	[0.001, 0.01]
	Group ^a^ Sex	0.088	0.13	0.658	[-0.17, 0.34]

^a^Estimated from frequentist methods, ʎ: Power transformation value estimated from Box-cox or Yeo-Johnson methods

### Associations between expression of genes and clinical data

**Fig. 3:**
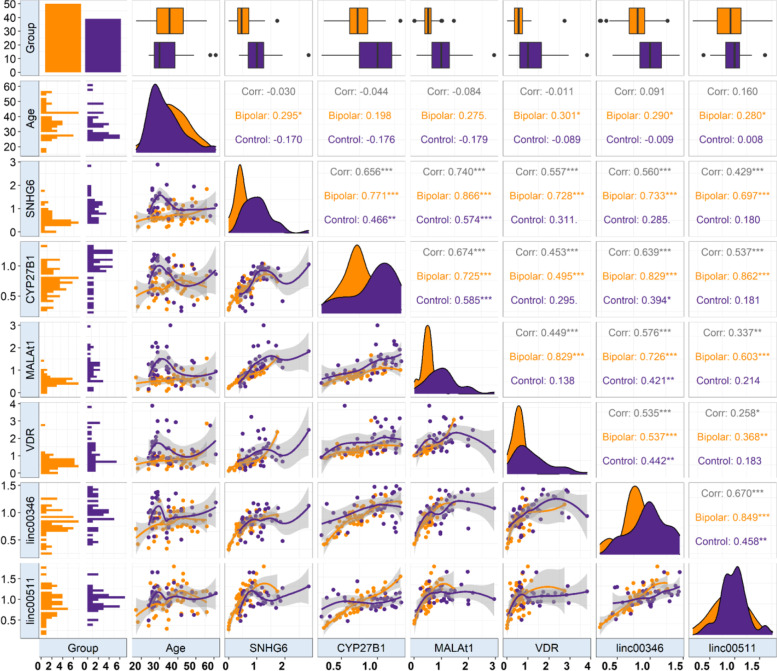
Correlations between expression of genes and age parameter as well as pairwise correlations between VDR-associated genes. The correlation coefficients in cases and controls are shown in orange and pink, respectively

Then, we gauged correlation between expression levels of genes in distinct study subgroups. Expressions of all genes except for *CYP27B1* were correlated with age of cases. Yet, expression of none of them was correlated with age of healthy controls. Significant correlations were demonstrated between all genes pairs among patients. However, among healthy controls several pairs of genes were not correlated (Fig. 3).

### ROC curve analyses

**Fig. 4:**
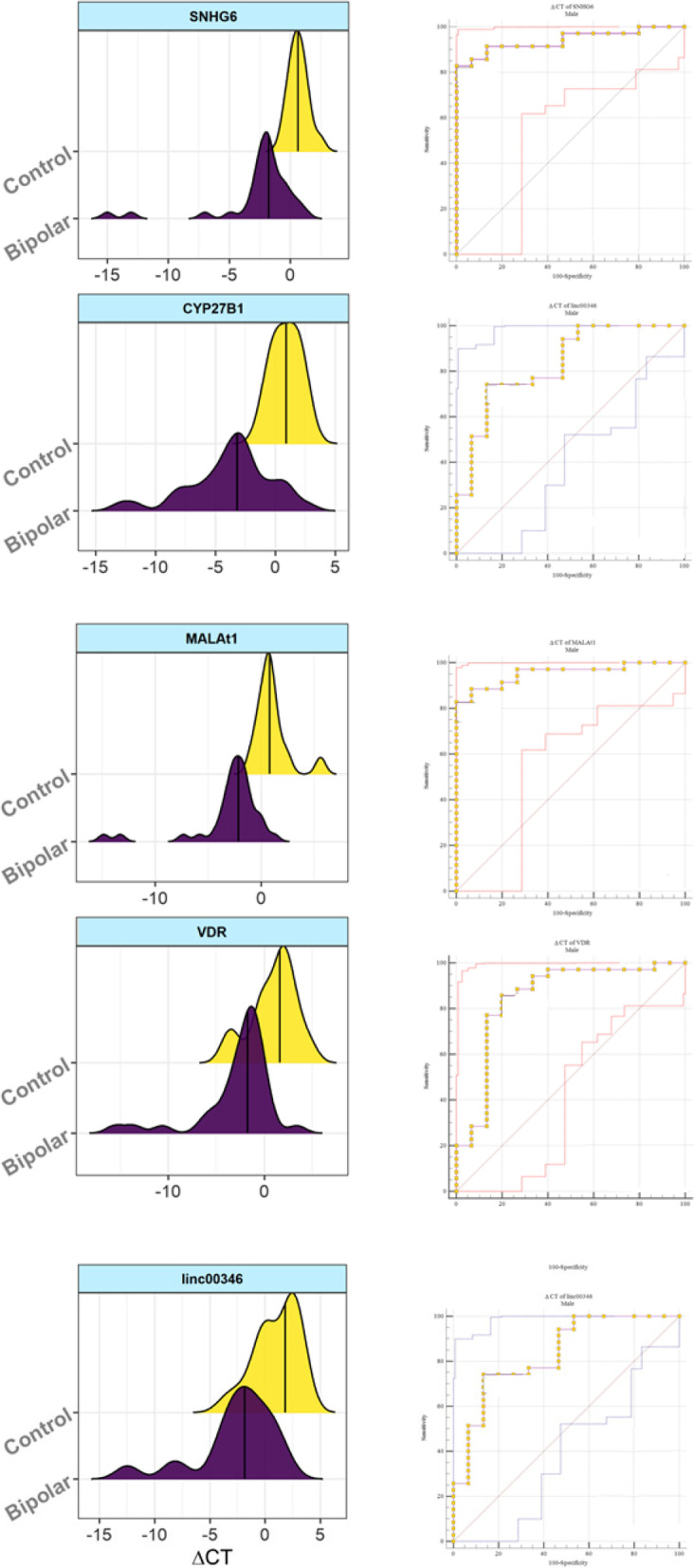
Diagnostic power of genes in separation of cases and controls. Right panels show the distribution of Delta Ct values of genes among patents (pink) and controls (yellow). Left panels show ROC curves and the obtained AUC, sensitivity and specificity values

Next, we appraised the power of genes in separating between cases and controls. *CYP27B1* had AUC, values of 0.58 and 0.91 among females and male, respectively. *SNHG6* had AUC value of 0.71 and 0.94 among females and males, respectively. *Linc00346* and *MALAT1* had AUC value of 0.83 and 0.95 among males, respectively. The AUC values of VDR were 0.85 and 0.74 among males and total subjects, respectively. Figure 4 shows the diagnostic power of genes in separating between cases and controls.

## Discussion

Several lines of evidence propose contribution of VDR signaling in the neurodevelopment and pathology of neurologic disorders. For instance, VDR is greatly expressed in the central nervous system and can modulate behavioral and emotional functions [22]. VDR has been shown to be expressed on the plasma membrane of neurons in conjunction with amyloid precursor protein, ADAM10 or Nicastrin [23]. So as to find the possible impact of VDR dysregulation in the development of BD, we examined expression of VDR-associated genes in these patients. Expression of *SNHG6* was significantly higher in both male and female cases compared with matched controls. Expressions of *CYP27B1*, *MALAT1* and *Linc00346* were up-regulated in total cases vs. controls and in male cases compared with male controls. This sex-based difference in expression of genes might be explained by the putative role of estrogen in modulation of gene expression. This assumption is reinforced by the observed effects of estradiol on expression of *MALAT1* in the context of endometriosis [24]. Expression of *VDR* was up-regulated in total cases compared with controls. Therefore, we have identified up-regulation of nearly all assessed genes from this pathway in male BD subjects. In a research conducted by Gezen-Ak et al. up-regulation of VDR and 25 hydroxy vitamin D3-24 hydroxylase might reflect “higher need for vitamin D” in hippocampus and possible effects of vitamin D shortage in cognitive defects and neurodegenerative processes [25]. Similarly, the detected up-regulation of VDR-associated genes in BD patients in the current study may indicate vitamin D deficiency in these patients.

We have also described over-expression of *VDR*, *CYP27B1*, and *CYP24A1* in peripheral blood of patients with schizophrenia compared with healthy subjects in our previous study. This finding is concordant with the reported overlap of genetic predisposing factors of schizophrenia and BD, as demonstrated by family, twin, and adoption studies [18].

Neuron-specific vitamin D signaling has also been shown to attenuate activation of microglial cells and induction of autoimmune responses in the central nervous system [26]. Immune deregulation has also been connected with the pathogenesis of BD through various routes including cytokine-induced alterations in the monoamine levels, induction of oxidative stress and aberrant activation of microglia [27, 28]. Therefore, the observed dysregulation of VDR-associated genes in the BD cases might also affect BD pathogenesis through altering immune responses.

In addition, VDR affects nuclear transcription and regulates levels of the dopamine receptor [13], thus influencing pathogenesis of BD through this route. Moreover, a number of VDR-associated lncRNAs have been shown to affect function of neurons. For instance, *SNHG6* can influence apoptosis of neurons through affecting miR‐181c‐5p/BIM signaling [29]. Most notably, miR-181 family has established role in the neurodevelopment [30]. *MALAT1* has been found to suppress neuronal apoptosis and neuroinflammatory responses while stimulating neurite outgrowth [31].

ROC curve analysis demonstrated high sensitivity and specificity of expression levels of *MALAT1*, *SNHG6*, *CYP27B1*, *VDR* and *Linc00346* in separation of male patients from male controls, indicating their putative function as circulatory markers for BD. Future assessment of their levels in distinct clinical phases of BD and appraisal of the influence of prescribed medications on expression of these genes would expand our knowledge about the biomarker role of these transcripts.

We also evaluated correlations between expression levels of genes in distinct study subdivisions. Expressions of all genes except for *CYP27B1* were correlated with age of patients. Yet, expression of none of them was correlated with age of healthy controls. Therefore, age affects expression of these genes in a disease-specific manner. Significant correlations were demonstrated between all genes pairs among patients. However, among healthy controls several pairs of genes were not correlated, indicating the influence of disease on construction of an interaction network between these genes. Alternatively, establishment of such interaction network might be implicated in the pathogenesis of BD.

In brief, the current study shows dysregulation of VDR-associated genes in BD and suggests these genes as possible markers for BD. These findings might provide new insight in the mechanistical approaches in BD and suggest novel targets for treatment of this disorder. Based on the complexity of VDR-associated signaling pathways and involvement of several non-coding RNAs as well as protein coding genes in these networks, future studies should focus on comprehensive appraisal of related genes through high throughput RNA sequencing strategies and validate these results in larger cohorts of patients.

### Strength and limitations of the study

Our study is among the first studies assessing the impact of VDR-related lncRNAs in the pathogenesis of BD in a homogenous population patients in terms of the type of administered medication. Our study has some limitations regarding sample size, lack of assessment of the effects of medications on expression of genes and levels of vitamin D. The sample size problem is more prominent in subgroup analysis by sex. Since the number of samples is even smaller in this type of analysis, this may lead to insufficient statistical efficiency. To rule out the impact of Carbamazepine on gene expression, it is necessary to include a group of drug-naïve patients. Moreover, it is recommended to assess vitamin D levels in both study groups to find possible effects of vitamin D insufficiency on expression of these genes and activity of related signaling pathways. Taken together, the findings of the current study should be interpreted carefully as the sample size in this study is small and the results have not been validated in another independent cohort. Moreover, this study was a candidate gene study that has no genome-wide context. Besides, we used peripheral blood tissue as a source for gene expression analysis and did not examine its relevance to brain.

## Conclusion

The current study suggests VDR-associated genes as possible markers for BD and warrants additional studies in this field to unravel the underlying mechanisms.

## Data Availability

The datasets used and/or analyzed during the current study are available from the corresponding author on reasonable request.
